# Aligning intuition and theory: enhancing the replicability of behaviour change interventions in cancer genetics

**DOI:** 10.1186/s43058-020-00054-0

**Published:** 2020-10-14

**Authors:** Natalie Taylor, Emma Healey, April Morrow, Sian Greening, Claire E. Wakefield, Linda Warwick, Rachel Williams, Katherine M. Tucker

**Affiliations:** 1 Cancer Research Division, Cancer Council New South Wales, Sydney, New South Wales 2011 Australia; 2 Faculty of Medicine and Health, University of Sydney, Camperdown, Sydney, NSW 2006 Australia; 3 Prince of Wales Clinical School, Faculty of Medicine, University of New South Wales, Randwick, New South Wales 2031 Australia; 4 Illawarra Cancer Care Centre, Wollongong Hospital, Wollongong, New South Wales 2500 Australia; 5 Kids Cancer Centre, Sydney Children’s Hospital, Sydney, New South Wales 2031 Australia; 6 School of Women’s and Children’s Health, Discipline of Paediatrics, UNSW Medicine, University of New South Wales, Sydney, NSW Australia; 7 ACT Genetic Service, The Canberra Hospital, Woden, Australian Capital Territory 2606 Australia; 8 Hereditary Cancer Centre, Prince of Wales Hospital, Sydney, New South Wales 2031 Australia

**Keywords:** Communication, Healthcare quality improvement, Implementation science, Quality improvement methodologies, Behaviour change techniques

## Abstract

**Background:**

Despite considerable encouragement for healthcare professionals to use or be clear about the theory used in their improvement programmes, the uptake of these approaches to design interventions or report their content is lacking. Recommendations suggest healthcare practitioners work with social and/or behavioural scientists to gain expertise in programme theory, ideally before, but even during or after the work is done. We aim to demonstrate the extent to which intuitive intervention strategies designed by healthcare professionals to overcome patient barriers to communicating genetic cancer risk information to family members align with a theoretical framework of behaviour change.

**Methods:**

As part of a pre-post intervention study, a team of genetic counsellors aimed to understand, and design interventions to overcome, the major barriers a group of familial cancer patients face around communicating hereditary cancer risk information to their relatives. A behavioural change specialist worked with the team to review and recode barriers and interventions according to the Theoretical Domains Framework (TDF) and 93 behaviour change techniques (BCTs). Resulting BCTs were cross-referenced against the Theory and Techniques Tool to examine whether evidence-based mechanistic links have been established to date.

**Results:**

Five themes emerged from the genetic counsellor coded barriers, which when recoded according to the TDF represented seven domains of behaviour change. Forty-five experiential and intuitive interventions were used to tackle key barriers. These were represented by 21 BCTs, which were found to be used on 131 occasions. The full mapping exercise is presented, resulting in a suite of intervention strategies explicitly linked to a theoretical framework. Structured, written reflections were provided retrospectively by the core clinical team.

**Conclusions:**

Although the ideal is to use theory prospectively, or even whilst a project is underway, making links between theory and interventions explicit, even retrospectively, can contribute towards standardising intervention strategies, furthering understanding of intervention effects, and enhancing the opportunities for accurate replicability and generalisability across other settings. Demonstrating to healthcare professionals how their intuition aligns with theory may highlight the additional benefits that theory has to offer and serve to promote its use in improvement.

Contributions to the literature

- Retrospective coding of intuitively designed genetic counselling interventions enabled the development of a standardised suite of strategies with explicit links to an underlying theoretical framework of behaviour change.
- Making these links explicit will enhance the replicability and generalisability of these intervention strategies within and beyond the cancer genetics setting, whilst also providing opportunities to progress current understanding about the mechanisms through which intervention components can contribute to patient behaviour change.

## Background

The demanding nature of healthcare professional roles in the clinical setting often necessitates the use of ‘on the spot’ intuition and experiential knowledge-based interventions to support patient behaviour change. Whilst this ‘informal theory’ is always at work in improvement, healthcare professionals are typically not aware of it or do not make it explicit [1]; this makes the replicability and generalisability of interventions problematic [2]. Consequently, those attempting to solve similar problems often waste valuable time and resources reinventing the wheel, without being able to learn from reliable accounts of the most effective intervention components for eliciting patient behaviour change and achieving improved outcomes [3, 4].

One recommendation for overcoming these issues is for healthcare practitioners to join forces with social and/or behavioural scientists to provide expertise in programme theory, ideally before, but even during or after the work is done [1]. This may help to address the recognised need to specify interventions in greater detail with their underlying theory and with more consistent terminology [5, 6], so that we can work towards identifying the mechanisms of action behind success [7].

One rapidly evolving field which requires healthcare professionals to carefully deliver patient-focused interventions in a time-critical fashion is that of cancer genetics. Genetic counsellors play a vital role in interpreting test results, informing patients of their genetic risks, and educating and counselling patients who potentially carry life-limiting genetic mutations [8]. The demand for these services is snowballing with each genetic discovery [9]. Genetic counsellors have a challenging role in encouraging patients to communicate hereditary cancer risk information to their blood relatives [10]—who need to be made aware of and make informed decisions about how to manage their own genetic cancer risks—often relying on clinical experience gained on the job and their intuition to support patients [11]. Factors including personality, skillset, experience, natural ability, and awareness can affect the extent to which genetic counsellors can work effectively with patients to ensure they disseminate information about these hereditary cancer risks to family members [11]. Whilst there is a body of literature focusing on factors affecting communication of genetic-related cancer risk between probands and their relatives [12, 13], and checklists to cover general risk communication with patients [14], evidence-based information to support genetics counsellors in identifying patient barriers to dissemination, and using appropriate strategies to help overcome these barriers is limited.

To tackle this problem, an Australian cancer genetics team used translational research funding to design and test a set of strategies they could use to assist patients with BRCA1 and BRCA2 gene mutations (predominantly increasing risk of breast, ovarian, and fallopian tube cancers) to identify and overcome information dissemination barriers [15].

Whilst some theoretical frameworks have been specifically designed to be accessible to those tasked with improvement and/or implementation in the healthcare system generally (e.g. the Theoretical Domains Framework: TDF) [16, 17], education and training opportunities to promote their widespread use amongst healthcare professionals are lacking. As a result, the majority remain unaware that such frameworks exist or lack the understanding and skillset to apply them [1]. This team—as is commonplace in healthcare improvement—relied on their intuition and experiential knowledge to undertake this task.

Nonetheless, working with healthcare professionals retrospectively in aligning these barriers and management strategies with a theoretical framework can facilitate intervention replication, tests for effectiveness, generalisability, and sharing of interventions within other realms of cancer genetics and broader areas of clinical practice. Furthermore, determining the extent to which the strategies used by the genetic counsellors are represented by existing behaviour change techniques (BCTs) [5] with established mechanistic links may help to optimise the design of prospective intervention strategies, whilst developing an understanding of the processes through which these strategies produce their effects [18]. Therefore, in this study, we aim to establish the extent to which:

1. Patient barriers to communicating BRCA1/BRCA2 risk to family members intuitively identified by healthcare professionals align with a theoretical framework of behaviour change;
2. Interventions designed intuitively by healthcare professionals to overcome patient barriers in communicating BRCA1/BRCA2 risk to family members align with evidence-based BCTs;
3. The intuitive barriers and corresponding intervention strategies align with theoretical behaviour change domains and corresponding BCTs that demonstrate evidence of mechanistic links.

## Methods

### Context: BRCA family dissemination pilot study

As part of a pre-post pilot study across four large hospitals in New South Wales, Australia, details of which are described elsewhere [15], the team aimed to understand the major barriers that BRCA1/BRCA2 patients face when trying to communicate hereditary cancer risk information to their relatives. Of the 165 families who participated, information had been disseminated to 81.1% of at-risk relatives. However, 87 families had at least one uninformed relative, with an average of 6.5 individuals uninformed. Larger, geographically diverse families reported greater difficulty informing all relatives about the BRCA risk information. During genetic counsellor telephone appointments (EH), barriers were captured then coded in consultation with the clinical team [15]. Interventions to overcome barriers were prescribed on the spot (annotated later with reference to barriers identified in the literature review); calls with patients were reviewed weekly for 6 weeks then every 3 weeks thereafter with the core team (clinical geneticist—KT; senior genetic counsellor—RW).

### Aligning behaviour change theory to barriers and interventions

A behavioural change specialist (NT) worked with the core team (EH, RW, KT) over four meetings (8 h) to retrospectively review in detail barriers and interventions from the study dataset. Patient barriers data were re-coded according to the TDF [5, 16], which classifies barriers according to theoretically underpinned psychosocial domains of behaviour change (e.g. knowledge: ‘*an awareness or existence of something*’; beliefs about capabilities: ‘acceptance of the truth, reality, or validity about outcomes of a behaviour in a given situation’; emotion: ‘a complex reaction pattern, involving experiential, behavioural and physiological elements, by which the individual attempts to deal with a personally significant matter or event’).

Intervention strategies designed to address barriers were mapped against a taxonomy of published BCTs and associated definitions [5] (e.g. social support (practical): ‘advise on, arrange, or provide practical help for performance of the behaviour’; anticipated regret: ‘Induce or raise awareness of expectations of future regret about the performance of unwanted behaviour’, credible source: ‘present verbal or visual communication of information from a credible source in favour of or against the behaviour’). All recoding was undertaken by NT (with specific training in and extensive experience of BCT coding), who consulted with the rest of the team to cross check the outcomes of the exercise.

Resulting BCTs were cross-referenced against the Theory and Techniques Tool [18]—an interactive online resource consolidating links between BCTs and their mechanisms of action (MoAs; represented in this study through the TDF domains) from the existing evidence base. The Theory and Techniques Tool is based on a comprehensive triangulation study [18] bringing together evidence from a literature synthesis of BCT-MoA links described in published intervention studies [19] and outcomes of a large expert consensus study rating BCT-MoA linkages [20]. The use of the tool allowed us to examine whether theoretical links have been established (to date) between the BCTs coded here and their corresponding MoA (according to TDF domain). Findings were reported in line with the TIDieR template for intervention description and replication (TIDieR) checklist and guide (Supplementary File 1) [21].

After study completion, the core clinical team were asked to provide a structured written reflection to describe how the exercise was (or was not) useful and whether or not it would change the way they approached the design of behaviour change interventions for improving clinical practice in the future.

## Results

### Aligning intuitively coded barriers to the TDF

Five themes emerged from the originally coded barriers by the genetic counsellor: ‘emotion’, ‘loss of contact’, ‘language or education barrier’, ‘dissemination responsibilities’, and ‘misunderstanding’. When recoded according to the TDF, seven psychological domains of behaviour change were represented: ‘emotion’, ‘environmental context and resources’, ‘skills’, ‘social role and identity’, ‘beliefs about consequences’, ‘knowledge’, and ‘social influences’. To provide some examples in context, the reported barrier of being ‘concerned about telling their child before they are old enough to cope with the information (protective parenting)’ was originally coded by the team as ‘emotion’ and aligned with the TDF domain of ‘emotion’; the reported barrier of ‘not realising that cousins would be at risk’ was intuitively coded as ‘misunderstanding’ and aligned with the TDF domain of ‘knowledge’; and the reported barrier of ‘immigration, separation/divorce/death of linking relative, estrangement, or general loss of contact’ was intuitively coded as ‘loss of contact’ and represented the TDF domain of ‘environmental context and resources’.

### Aligning intuitively generated intervention strategies to BCTs

A total of 45 different intuitive interventions were used by the genetic counsellor to tackle key barriers raised by patients. These were represented by 21 BCTs, which were found to be used on 131 occasions (‘occasions’ refer to any instance a BCT was identified in an intuitively described intervention strategy, noting that each intervention strategy can contain multiple BCTs, and one BCT can be present in multiple different intervention strategies). Table 1 demonstrates how a selection of the originally coded key barriers to dissemination aligned with the TDF and how the interventions prescribed mapped against BCTs. Supplementary File 2 provides the results of the full coding exercise. For example, for a patient who doesn’t have contact with their relatives, the intuitively generated intervention was to (a) ‘suggest they ask a linking person (e.g. aunt/uncle/family friend)’ and this represented the BCT ‘Provide instruction on how to perform the behaviour’ (advice or agree on how to perform the behaviour), and (b) ‘discuss that some patients have been successful using the phonebook, Facebook or ancestry.com to get in touch with long-lost-relatives’, and that ‘some patients have even reported BRCA to be the catalyst to contact and reunite with relatives, and it has brought their family closer’. These strategies represented the BCTs of ‘Information about social and environmental consequences’ (provide information—e.g. written, verbal, visual—about social and environmental consequences of performing the behaviour); ‘social support (practical)’ (advise on, arrange, or provide practical help for performance of the behaviour); ‘vicarious consequences’ (prompt observation of the consequences for others, such as rewards and punishments, when they perform the behaviour), and ‘social comparison’ (draw attention to others’ performance to allow comparison with the person’s own performance).

**Table 1:**
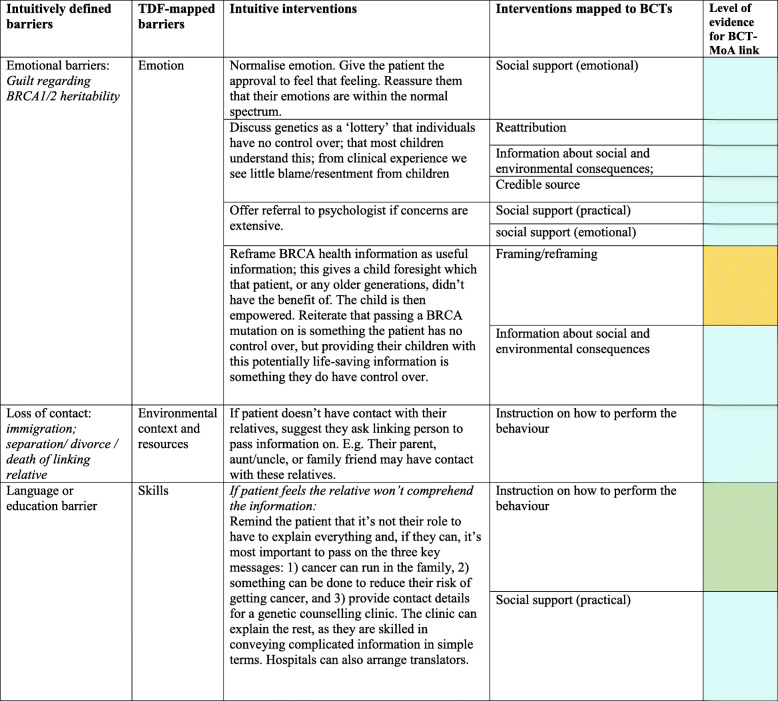

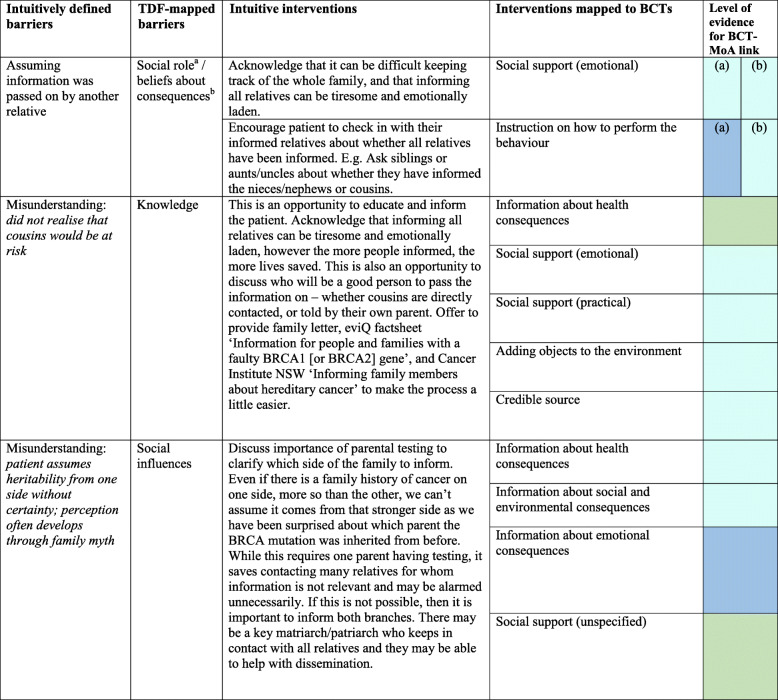
Example of mapping intuitively derived barriers and interventions to the Theoretical Domains Framework domains and behaviour change techniques (BCTs)

Corresponding *p* values for literature-established links between BCTs and their mechanisms of action are available elsewhere (see Carey et al. 2018) and online via the link below: https://theoryandtechniquetool.humanbehaviourchange.org/

Supplementary file 2 provides the results of the full coding exercise

*BCT* behaviour change technique, *MoA* mechanism of action, *TDF* Theoretical Domains Framework

Of the 21 BCTs represented, the most frequently used were ‘credible source’ (represented in 17 intuitive interventions), ‘social support - practical’ (represented in 16 intuitive interventions), ‘social support - emotional’ (represented in 13 intuitive interventions), and ‘information about health consequences’ (represented in 13 intuitive interventions).

The ‘emotion’ domain used the largest number of interventions (33) and BCTs (21 were used across 93 occasions), followed by ‘knowledge’ (interventions = 3; BCTs = 8 used across 14 occasions), ‘environmental context and resources’ (interventions = 3; BCTs = 7 used across 8 occasions), ‘skills’ (interventions = 2; BCTs = 5 used across 6 occasions), ‘social role and identity’ or ‘beliefs about consequences’ (interventions = 3; BCTs = 6 used across 6 occasions), and ‘social influences’ (interventions = 1; BCTs = 4 used across 4 occasions).

### Establishing the extent to which intuitive barriers and corresponding intervention strategies aligned with evidence-based TDF domains and corresponding BCTs

After cross-referencing against the Theory and Technique Tool [18], we found that eight of the 21 BCTs identified through this coding exercise were used to address a corresponding TDF coded barrier that has previously demonstrated statistically significant mechanistic links (i.e. theoretical alignment agreed upon by expert consensus AND associations in the intervention literature synthesis), and this occurred on 13/131 (10%) occasions: ‘reduce negative emotions’ (TDF domain = ‘emotion’), ‘social support – practical’ and ‘adding objects to the environment’ (TDF domain = ‘environmental context and resources’), ‘instruction on how to perform the behaviour’ (TDF domain = ‘skills’), ‘anticipated regret’ and ‘salience of consequences’ (TDF domain = ‘beliefs about consequences’), ‘information about health consequences’ and ‘instruction on how to perform the behaviour’ (TDF domain = ‘knowledge’), and ‘social support – unspecified’ (TDF domain = ‘social influences’) [1].

Six of the 21 BCTs were found to be ‘non-links’ (e.g., BCT-MoA link absent in literature synthesis AND experts in consensus study agreed there was no theoretical link), used on 9/131 (7%) of occasions. However, four of these occasions were in the context of interventions with multiple BCTs coded (as is commonplace in BCT coding exercises), where at least one of the accompanying BCTs had evidence of mechanistic links (as defined above). One occasion was accompanied by a BCT with ‘inconclusive’ evidence (Theory and Technique Tool triangulation results = 69% agreement with BCT-MoA link but failed to meet the 80% study threshold). The remaining 3/131 (2%) occasions were ‘instruction on how to perform the behaviour’ (TDF domain = ‘emotion’; used on two occasions) and ‘problem solving’ (TDF domain = ‘emotion’; used on one occasion).

The remaining BCTs had either an absence of evidence to draw conclusions about mechanistic links [16 BCTs used on 95/131 (73%) occasions] *or* existing evidence from literature and/or expert consensus was deemed ‘inconclusive’ [5 BCTs used on 14/131 (11%) occasions]. In some instances, the same BCT represented a ‘link’ on some occasions, and a ‘non-link’ on other occasions, depending on the TDF domain being mechanistically targeted (hence BCT values above exceed the total). For example, when used to address the TDF domain ‘knowledge’ or ‘skills’, the BCT ‘instruction on how to perform the behaviour’ represents a mechanistic link, but when used to address the TDF domain ‘emotion’, represents a non-link. Supplementary File 2 provides the levels of evidence for mechanistic links for all 131 occasions, as derived from the Theory and Techniques Tool.

### Healthcare provider reflections

All core clinical team members (*n* = 3) provided a written retrospective reflection on their experience of the coding exercise, key learnings, perceived generalisability, intentions for further use, and training and support needs. All three clinical team members felt it was a useful exercise and could see value in continuing to apply implementation science and behaviour change theory in their ongoing clinical and/or research practice. Responses have been categorised and summarised in Table 2.

**Table 2:** Healthcare provider reflections

Category	Illustrative quotes
**Benefits of the coding exercise and key learnings**	‘Personally I found the coding exercise incredibly useful in that it forced me to think in the structured implementation science way and particularly the implementation barriers allowed me to generalise to other services we are introducing.’ Team Member 1
	‘The coding allowed us to identify and more clearly describe how the intuitive interventions helped to initiate behaviour change (dissemination).’ Team Member 2
	‘The intuitive approaches were initially formulated by contemporary clinical practice informed by many studies. However, these were unstructured. This coding has enabled a structured manual for future staff and existing staff. Having a small number of codes to consider interventions for, was far less daunting, and made a lot of sense. It has given me a greater appreciation of how barriers can be more readily identified, and interventions put in place.’ Team Member 3
	‘Whereas we have not continued with the structured patient follow-up calls as in this study due to increased referrals/workload, the staff are motivated to understand identification of these barriers and what interventions to apply.’ Team Member 3
**Replicability of coded interventions**	*‘*These interventions came about intuitively and the study interviews were all conducted by the one researcher (myself). So from a research perspective, recording them in the coding table provides consistency if further research is to be conducted (or multiple researchers are to work with families). And from a clinical perspective, we now have a practical manual on how to support families with dissemination challenges, and this may be useful for both current genetic counsellors and as training for new genetic counsellors.’ Team Member 2
	‘This coding has enabled a structured manual for future staff and existing staff. One such practical example was the ad hoc nature of each of the genetic counsellors having their own incomplete lists of international genetics contacts. <The study genetic counsellor> collated these from far and wide outside of the study hospitals to produce an excellent resource which continues to be used and added to.’ Team Member 3
**Perceived generalisability**	‘While the interventions were used on a specific BRCA cohort, the same theories are applicable to other familial cancer conditions and other genetic conditions. At this stage the process has not been used to address other clinical problems, but could potentially be used to consider how to motivate patients to overcome other barriers, such as reluctance to have a risk-reducing surgery, breast screening, colonoscopy, or compliance issues with risk-reducing medication such as tamoxifen or aspirin.’ Team Member 2
	‘The intervention strategies helped to reassure us we were going ok and it allowed us to generalise the interventions so other services can pick up on what we were doing that worked.’ Team Member 1
**Future use of implementation science and behaviour change theory**	‘We addressed the problem of not getting appropriate referrals of CRC patients with abnormal immunohistochemistry and set about approaching the problem with implementation science research questions – this has led to a randomised trial.’ Team Member 1
	‘I found that after coding so many I was starting to be able to see trends in my day to day work and now think about implementation before I introduce new guidelines. One of the disadvantages is the time it took and the sinking feeling that we had done all this work without having accessed the implementation scientists earlier!’ Team Member 1
	‘I personally think about how I am responding to a new problem with a patient by thinking about the TDF and it helps me put a framework around how I approach it.’ Team Member 1
	‘I am thinking about this in other research and clinical problem areas. We can currently consider this with other studies, such as our newsletter implementation for CRC/polyposis cohort-planned 2020, and our CONTACT study -genetic counselling via telehealth currently completing its pilot before the RCT roll out.’ Team Member 3
**Training and support needs**	‘Using behaviour change theory in the future, or more widely amongst the genetic counselling profession, would require some training and input from staff with prior experience. The process/theory is not common knowledge, but is very practical and useful in application.’ Team Member 2
	‘Training and support is needed for clinical staff. Although when I've done this, it is very straight forward, I still find the lines blurry differentiating between identifying the problem (coded) and then applying the intervention.’ Team Member 3

## Discussion

Retrospective application of behaviour change theory against intuitively derived behaviour change interventions by a multidisciplinary team of clinicians and behavioural psychologists is possible. Although the ideal is to do this work prospectively and throughout, the early application of formal theory can appear an abstract and intimidating task for healthcare professionals [4]. Perhaps a more realistic starting point is to ‘demystify’ theory by demonstrating how their intuition aligns with a theoretical framework and to give credit for successful improvement where credit is due.

Cross-referencing the BCTs represented in intuitive genetic counsellor strategies against the Theory and Techniques Tool highlighted a proportion of strategies with existing evidence-based links between BCTs and their corresponding MoAs [18]. Where such links exist, these provide a possible explanation for the processes through which these strategies produce their intended effects and may inform the design of future interventions to enhance opportunities for success. For example, where multiple intuitive interventions exist for a given dissemination barrier, the genetic counsellor can refer prospectively to the levels of evidence in the coded resource (i.e. Supplementary file 2) to select in favour of interventions with established links. Where established links do not yet exist, the Theory and Techniques Tool could also be used to generate new intervention strategies according to evidence-based BCT-MoA links.

This exercise may also generate a smoother pathway for demonstrating how healthcare professional intuition does not align with theory and how resulting interventions may have been ineffective or counterproductive. For example, providing contextually relevant examples to demonstrate the ‘non-links’ that exist (and why) between intuitively selected BCTs against corresponding MoAs (e.g. ‘instruction on how to perform the behaviour’ to address the TDF domain ‘emotion’ in the context of BRCA risk communication) may serve to demystify theory for healthcare professionals and promote its use in prospective intervention design.

Furthermore, for the most frequently represented BCTs (e.g. credible source, emotional support-practical), such links have not yet been established due to lack of evidence. This highlights areas where specific tests of effectiveness may help to establish a BCT-MoA link (or non-link), hence contributing to the implementation science literature and allowing further development of the Theory and Techniques Tool [18]. Given the more frequent use of these techniques in this context (i.e. communicating risk information for BRCA 1/2 in cancer genetics), demonstrating effectiveness may also help to generate a case for use of these particular techniques for communicating risk across other genetic mutation types (e.g. Lynch syndrome, Li Fraumeni syndrome) [22, 23].

Through this process, we have produced a standardised suite of interventions matched to key psychosocial barriers, identified which BCTs are being more frequently represented through intervention strategies in this particular context of cancer genetics, and established the extent to which intuitive barriers and corresponding intervention strategies align with TDF domains and corresponding BCTs that have demonstrated evidence of mechanistic links. The core clinical team found the process to be highly valuable in developing a practical understanding of the ways in which behaviour change theory can be applied to intervention design. Some have since applied implementation science and/or behaviour change theory principles to their clinical and research practices (e.g. [24, 25]).

Explicitly stating the underlying theory for each of these intervention strategies will provide opportunities for learning by enhancing opportunities for (a) understanding more about the intervention components that are or are not contributing to behaviour change (i.e. in this case increased risk communication by probands to relatives) and (b) refining intervention strategies according to specific BCT definitions to maximise impact and allow specific testing of theoretically underpinned interventions that could be used to address similar psychosocial barriers in the cancer genetics context (e.g. Lynch syndrome, Cowden syndrome, Li Fraumeni syndrome) [22, 23] and possibly for other healthcare conditions and contexts (e.g. cardiovascular disease, neuromuscular disorders, cognitive impairment). Generating this kind of evidence could support healthcare professionals to select effective strategies for achieving patient behaviour change and translating cancer genetics evidence into practice.

Whilst this study has explored a novel concept around the alignment between intuition and theory and produced a resource matching standardised behavioural change techniques to what is intuitively generated by professionals in the field, there are several limitations that should be noted. First, the efficacy of the BCTs in terms of improving patient communication has not been demonstrated, nor has the efficacy of the retrospective mapping analysis on influencing practices of healthcare providers. Testing the impact of these interventions in the local setting would help to generate a solid foundation from which we can build on to prospectively modify existing and design new interventions using theory, test these using controlled methods, and further understand key mechanisms of change. Second, we were unable to formally evaluate *if* and *how* the intervention strategies would differ if theory were to be used prospectively. However, further work is underway in the context of hereditary cancer clinical practice to directly test and explore the differences in effects of intuitively and theory informed approaches to intervention design [24, 25].

## Conclusion

Whilst there has been considerable encouragement for healthcare professionals to use or be clear about the theory used in their improvement programmes, the uptake of these approaches to design interventions or report their content is still lacking. Whilst the goal remains for theory to be used prospectively, retrospective mapping may illustrate to healthcare professionals the additional benefits that theory has to offer (e.g. guiding intervention design; opportunities for intervention replicability; translation to other settings; identifying mechanisms of change; accurate tailoring of interventions; and longer-term time, resource, and cost savings), particularly when used from the outset of an improvement or implementation project. Reflecting on their experiences, this small team of clinicians found value in the theory-mapping exercise and have since sought to apply key learnings in both their clinical and research practices, though highlighted a need for training and support. Further understanding healthcare perspectives on the results of such theory-intuition alignment mapping exercises will be an important avenue for enhancing approaches to co-design of behaviour change interventions for improving clinical practice.

## Supplementary information

**Additional file 1:** Supplementary File 1. The TIDieR (Template for Intervention Description and Replication) Checklist.

**Additional file 2:** Supplementary File 2. Mapping intuitively derived barriers and interventions to the TDF and BCTs.

## Data Availability

All data generated or analysed during this study are included in this published article [and its supplementary information files.

## Abbreviations

BRCA1: Breast cancer 1, early onset
BRCA2: Breast cancer 2, early onset
TDF: Theoretical Domains Framework
BCTs: Behaviour change techniques
MoAs: Mechanisms of action
TIDieR: Template for Intervention Description and Replication
RCT: Randomised controlled trial
CRC: Colorectal cancer
