# Supplementary material for: Aligning intuition and theory: enhancing the replicability of behaviour change interventions in cancer genetics
Source: Implement Sci Commun. 2020 Oct 14;1:90. doi: 10.1186/s43058-020-00054-0 (PMC7557091; doi:10.1186/s43058-020-00054-0)
Supplement: Supplementary file 2 — Additional file 2: Supplementary File 2. Mapping intuitively derived barriers and interventions to the TDF and BCTs. [file 43058_2020_54_MOESM2_ESM.docx]

Supplementary file 2. Mapping intuitively derived barriers and interventions to the Theoretical Domains Framework domains and behaviour change techniques (BCTs), and illustrating the level of evidence for BCTs that demonstrate a mechanistic effect on specific TDF domains (i.e. the MoA).

| **Intuitive barriers** | **TDF barriers/MoA** | **Intuitive interventions** | **Interventions mapped to BCTs** | **Level of evidence for BCT-MoA link** | |
| --- | --- | --- | --- | --- | --- |
| Emotional barriers: *Guilt regarding BRCA1/2 heritability* | Emotion | Normalise emotion. Give the patient the approval to feel that feeling, and reassure them that their emotions are within the normal spectrum. | Social support (emotional) |  | |
|  |  | Discuss genetics as a ‘lottery’ that individuals have no control over; that most children understand this; from clinical experience we see little blame/resentment from children. | Reattribution |  | |
|  |  |  | Information about social and environmental consequences |  | |
|  |  |  | Credible source |  | |
|  |  | Offer referral to psychologist if concerns are extensive. | Social support (practical) |  | |
|  |  |  | social support (emotional) |  | |
|  |  | Reframe BRCA health information as useful and empowering; gives a child foresight which that patient, or older generations, didn’t have the benefit of. Reiterate that passing a BRCA mutation on is beyond the patient’s control, but providing their children with this potentially life-saving information is something they do have control over. | Framing/reframing; |  | |
|  |  |  | Information about social and environmental consequences |  | |
|  |  | Provide hope by explaining that risk-reducing options and fertility options will improve in the future when their child reaches a higher cancer risk period of their lives (30s onwards). | Information about health consequences; |  | |
|  |  |  | Information about social and environmental consequences |  | |
|  |  | Discuss that the patient themselves may have reduced distress after informing their child/relative; explain that current perspective might cause distress, prompt change in perspective by reframing the benefits of sharing the BRCA risk information – feelings of liberation, assistance with medical decision making from relatives who share the burden, and increased emotional support via shared experiences of the condition. | Information about emotional consequences; |  | |
|  |  |  | Framing/reframing; |  | |
|  |  |  | Information about social and environmental consequences; |  | |
|  |  |  | Reduce negative emotions |  | |
|  |  | Provide or direct the patient to resources which may reduce sense of guilt: a) Pink Hope website - this features stories of other BRCA carriers, which the patient may relate to; b) Printed or PDF brochures for information: including eviQ factsheet ‘Information for people and families with a faulty BRCA1 [or BRCA2] gene’ Cancer Institute NSW ‘Informing family members about hereditary cancer’, Royal Marsden NHS Foundation booklet ‘A Beginner’s Guide to BRCA1 and BRCA2’ and FORCE booklet ‘Talking About BRCA in Your Family Tree’. | Credible source; |  | |
|  |  |  | Social support (emotional); |  | |
|  |  |  | Information about health consequences |  | |
| Emotional barriers: *Concern of emotionally burdening relatives (e.g. relatives have enough on their plate)* | Emotion | Discuss that the burden of BRCA risk information is far less than the burden of a cancer diagnosis, which could be avoided. E.g. cancer diagnosis leads to ill-health, intensive treatment, time off work, potential loss of income, fear of recurrence and mortality. Discuss previous experiences of other families where individuals have tried to ‘protect’ relatives by not disclosing the BRCA risk information, resulting in that relative being diagnosed with cancer. This has led to anger and resentment from the relatives, and terrible guilt for the patient. | Comparative imagining of future outcomes; |  | |
|  |  |  | Vicarious consequences; |  | |
|  |  |  | Anticipated regret; |  | |
|  |  |  | Social comparison |  | |
| Emotional barriers: ***Practical*** *and/or geographical concerns (e.g. relatives live overseas and may not have a genetic service in their region* | Emotion | Compile a list of contact details for other genetic services in Australia and internationally. Elicit where the patient’s relatives are based, and offer to provide the relevant contact details of genetic counselling services in the relative’s geographical region. | Adding objects to the environment; |  | |
|  |  |  | Social support (practical) |  | |
|  |  | Explain availability of telehealth to support access to the health system for relatives living in remote areas. This aims to help the patient feel more empowered that they can direct their relatives ‘where to go’. | Social support (unspecified) |  | |
|  |  | Inform the patient that even if genetic services don’t exist in their relatives’ country, providing the information is still important because: 1) relatives can still access risk management (appropriate screening/consider risk-reducing surgery) even if they haven’t had testing to clarify mutation status; and 2) knowing this information might encourage symptom awareness. Provide eviQ factsheet ‘Information for people and families with a faulty BRCA1 [or BRCA2] gene’, which provides basic risk-management information and states: ‘If you decide not to have genetic testing, risk management is still recommended’. | Framing/reframing; |  | |
|  |  |  | Credible source; |  | |
|  |  |  | Information about health consequences; |  | |
| Emotional barriers: *concern about potential* ***medical*** *burden (e.g. relatives are already dealing with another medical/genetic diagnosis)* | Emotion | Show understanding for their hesitancy. | Social support (emotional) |  | |
|  |  | Negotiate a gradual approach to information dissemination; the patient could start by informing one relative (e.g., the easiest person they think they could tell). This may reduce sense of pressure, yet there is still plan to work towards more challenging communication situations in the near future. Also explain that if the first relative they inform reacts calmly and shows appreciation for the information, this may be positive reinforcement to tell others. | Graded task |  | |
|  |  |  | Information about social and environmental consequences |  | |
|  |  |  | Information about emotional consequences |  | |
|  |  | Explore patient perception about how their relative (who is dealing with health issues) coped when informed about the first diagnosis. a) After coping with the emotions, did they acknowledge the benefit of early diagnosis? If so, discuss that their relative may be similarly grateful for BRCA risk information in time. b) Was the relative’s poor coping related to the limited options available for treatment/cure? Acknowledge this as a normal response to the challenges of a potentially life-limiting illness. Discuss that a cancer predisposition differs in that there is a preventative focus (may provide eviQ factsheet ‘Information for people and families with a faulty BRCA1 [or BRCA2] gene’) and risk-reducing management options are available. Therefore, the relative may cope much better with this information. Consider use of analogy, such as avoiding a situation where ‘all the wheels have fallen off the bus’: i.e. the first health issue being the first wheel to fall off, and then a potentially preventable cancer diagnosis being the second wheel. | Comparative imagining of future outcomes; |  | |
|  |  |  | Information about health consequences; |  | |
|  |  |  | Information about social and environmental consequences; |  | |
|  |  |  | Salience of consequences; |  | |
|  |  |  | credible source |  | |
| Emotional barriers: *concern about* ***financial*** *implications(e.g. concern of cost for relative to see genetic service or have testing)* | Emotion | Discuss that in Australia, cost of appointment and testing can be covered by the public health system. Some other countries are similar – UK, Canada. For relatives in a ‘user-pays’ country, explain most information about BRCA testing quote the cost of initial mutation search ($1000-$3000)*, but their relative only needs to have a predictive test, which is usually a quarter/third of this cost. | Information about social and environmental consequences; |  | |
|  |  |  | Credible source |  | |
|  |  | Normalise concerns about insurance implications. Explain that a) Health insurance in Australia is not affected by BRCA testing outcome, and b) life insurance companies will only require information on genetic testing if an individual is taking out a new policy or changing an existing one.* Offer patient insurance factsheet from Centre for Genetics Education – ‘Life Insurance Products and Genetic Testing in Australia’. Also provide contact number for the Financial Services Council so the patient can seek their own advice. | Social support (practical); |  | |
|  |  |  | Credible source |  | |
|  |  | *Concern relatives will not be able to find a life-partner because of their* *own or another relatives experience*? Genetic counsellor can explain that this is often the exception rather than the rule, and stigma has lessened significantly over last few years (especially since Angelia Jolie announced her mutation status). If patient is fixated on this, recommend psychologist. | Information about social and environmental consequences; |  | |
|  |  |  | social support (emotional); |  | |
|  |  |  | Social comparison |  | |
|  |  | *Concern relatives will not be able to find a life-partner due to the increased risk for cancer diagnosis?*  Normalise that no partner is perfect. Explain that all individuals have genetic risk factors; their family are just one of the few who know what that risk factor is and can take control of it. Many partnerships face hardships and require support, whereas these partners will be in a unique position to be educated and prepared for these hardships. Additionally, risk of a cancer diagnosis may be lower than the average woman for relatives who take up risk-reducing surgical options. | Social support (unspecified); |  | |
|  |  |  | Framing/re-framing |  | |
|  |  | *Concern relatives will not be able to find a life-partner due to body image changes after risk-reducing surgeries*? Explain that both aesthetic and functional outcomes of risk-reducing surgeries have improved significantly over time. Connect the patient with another BRCA carrier who has undergone surgery and can provide personal experience. Encourage patient to attend annual BRCA1/BRCA2 information day to hear a talk given by a reconstructive surgeon, which includes see images of reconstructive outcomes. Encourage patient to visit online community forums such as Pink Hope. | Information about health consequences; |  | |
|  |  |  | Social support (practical); |  | |
|  |  |  | Social support (emotional); |  | |
|  |  |  | Salience of consequences; |  | |
|  |  |  | Credible source |  | |
|  |  | *Concern relatives will not be able to find a life-partner due to eviQ recommendations for women to have ovaries and fallopian tubes ≤40 years old.*  Acknowledge that some young women do feel greater urgency to find life-partner given recommendation of gynaecological surgery, however surgical timing is generally in line with declining fertility that average women face. Also explain that the family cancer clinics work with each individual to ensure the patient’s priorities are respected (i.e. some individuals may still have a child at 40-42 years old before having ovaries and fallopian tubes removed). | Information about health consequences |  | |
|  |  | *Concern relatives will not be able to find a life-partner due to religious or cultural concerns (often related to being a good stock for procreation)?*  Encourage patient to seek support, guidance (and hopefully reassurance) from their religious leader. | Social support (unspecified |  | |
| Emotional barriers: *Concern relative will not cope* | Emotion | Explore the patient’s relationship with their relative and the frequency of contact. Explore relatives’ previous ways of coping when they have received bad news. Discuss variations in coping styles, and*‘perceived’* vs *‘actual'* coping, i.e. relatives may become emotional talking about BRCA, but these occasions may be the times they want the patient’s emotional support (a coping mechanism), buton a day-to-day basis they are coping well. Also, if the relative is a close relative (e.g., child), suggest integration of psychologist or their GP (especially for relatives with a disability or mental health condition). | Framing/reframing; |  | |
|  |  |  | Information about social and environmental consequences; |  | |
|  |  |  | Social support (emotional); |  | |
|  |  |  | Social support (practical) |  | |
|  |  | Offer appointment to family – the patient can plan to tell their relative within the week before the appointment date. This provides the patient with a safety net and professional support, in case the relative copes poorly and has many worries and questions | Action planning; |  | |
|  |  |  | Social support (practical); |  | |
|  |  |  | Social support (emotional) |  | |
|  |  | Provide printed or PDF brochures for information, including: Cancer Institute NSW ‘Informing family members about hereditary cancer’, Royal Marsden NHS Foundation booklet ‘A Beginner’s Guide to BRCA1 and BRCA2’ and FORCE booklet ‘Talking About BRCA in Your Family Tree’. | Information about health consequences; |  | |
|  |  |  | Credible source; |  | |
|  |  |  | Social support (practical) |  | |
|  |  | *Practical coping problems, i.e., concern of needles:* Discuss the benefit of informing relatives for the purpose of knowledge for screening. These relatives can delay testing, as it is possible that saliva testing may be available in the near future. | Information about health consequences |  | |
| Emotional barriers: *Concerned about telling their child before they are old enough to cope with the information (protective parenting)* | Emotion | Discuss literature suggesting that children cope better when information is introduced at younger age (option to provide an analogy of adoption and the benefits of early age disclosure). This normalises the information and becomes part of child’s health history e.g. patient could say “mummy gets extra checks on her breast to makes sure she stays well, and when you’re older the doctor might do extra checks for you too”. Children feel more valued by being included, thus it can bring families closer. If the discussion is left until later age it can a) be more confronting b) cause resentment that information was kept secret, and c) create sense of a sudden rush into screening and urgency to have children (if delayed until aged 30s). | Credible source; |  | |
|  |  |  | Information about emotional consequences; |  | |
|  |  |  | Information about social and environmental consequences; |  | |
|  |  |  | Salience of consequences |  | |
|  |  |  | Instruction on how to perform the behaviour; |  | |
|  |  |  | Comparative imagining of future outcomes; |  | |
|  |  |  | Anticipated regret |  | |
|  |  | *Uncertainty about what to say/when to say it:*  Offer to role play. Discuss what information is important: a) basic information that can build over time, b) reassurance that BRCA mutation does not always lead to cancer and things can be done to reduce cancer risk, and c) recognise different needs of siblings depending on age/interest level. Discuss when to talk to children: easier to discuss informally while driving, cooking, or going on a walk together, rather than having a family sit down ‘intervention style’. Provide the Royal Marsden NHS Foundation booklet: ‘A Beginner’s Guide to BRCA1 and BRCA2’ and FORCE booklet ‘Talking About BRCA in Your Family Tree’, for explanation of how to explain genetic testing to children of different ages. | Behavioural practice/rehearsal; |  | |
|  |  |  | Instruction on how to perform the behaviour; |  | |
|  |  |  | Social support (practical); |  | |
|  |  |  | Credible source; |  | |
|  |  |  | Information about health consequences |  | |
|  |  | Offer appointment to family – once an appointment date is set, the patient can plan to tell their child the week before; then the parent can bring the family to the appointment. This provides a safety net if the child has questions and provides an opportunity for the child and genetic counsellor/geneticist to establish positive relationship for the future. | Action planning; |  | |
|  |  |  | Social support (practical); |  | |
|  |  |  | Social support (emotional) |  | |
| Emotional barriers: *Fear of a broken down relationship* | Emotion | *Fear that the relative will blame them:*  Discuss that anger can be a normal response that isn’t necessarily directed at the patient. Suggest patient explains reasons for disclosure (e.g. right to know, health benefits). Patient to inform relative that it is there choice whether or not to have testing and their right ‘not to know’ (i.e. not have testing) will be respected. Provide them with the Cancer Institute NSW ‘Informing family members about hereditary cancer’ leaflet (which addresses ‘what can I do if a family member reacts angrily and makes me feel uncomfortable?). Alternatively, offer family letter to relatives and suggest that patient can say “the clinic insisted that I pass this information onto you” to shift onus. | Social support (emotional); |  | |
|  |  |  | Instruction on how to perform the behaviour; |  | |
|  |  |  | Credible source |  | |
|  |  |  | Social support (practical); |  | |
|  |  |  | Adding objects to the environment |  | |
|  |  | *Fear is that nieces/nephews don’t know but patient doesn’t want to disrespect parental role of their sibling:*  Consider alternative dissemination methods. a) Explore whether patient has a good relationship with their sibling-in-law. This individual may be more receptive about the benefit of the BRCA risk information than their sibling, and thus ensure their children (the patient’s nieces/nephews) are informed. b) Alternatively, it is possible that adult cousins are going to give less thought to this, and may comfortably communicate between each other. Thus the patient might feel they can ask their own offspring (if an adult) to pass information on directly. | Problem solving; |  | |
|  |  |  | social support (unspecified) |  | |
| Family rift leads to active non-disclosure *(i.e., deliberate withholding from relatives particularly when there has been a family rift and they have little contact with the relative.)* | Emotion | Normalise the difficulty of estrangement, and their hesitation to make contact with this relative. Give the patient the time and freedom to vent their frustrations so that they feel heard, and rapport is maintained. If the patient hasn’t informed other relatives (with whom he/she is in comfortable contact with), focus on this first. Discussing dissemination to the estranged relative can be delayed until rapport is stronger**. | Social support (emotional); |  | |
|  |  |  | Graded task |  | |
|  |  | Discuss the patient’s sense of responsibility and empathy towards the next generation, who may be negatively affected if the BRCA risk information is not passed on (for example, the patient may have extreme anger/hatred towards sibling, but may be motivated to inform because of the concern for their nieces/nephews). | Salience of consequences; |  | |
|  |  |  | Anticipated regret; |  | |
|  |  |  | Comparative imagining of future outcomes |  | |
|  |  | Prompt patient to consider prospect of relative getting a diagnosis that could have been avoided. Consider use of analogy: ‘if you knew your relative’s car has bald tyres, wouldn’t you rather warn them of this, than wait for them to have an accident?’, or ‘if you knew a plane your relative was about to board had a 50% chance of crashing, would you still let them get on?’ | Salience of consequences; |  | |
|  |  |  | Anticipated regret; |  | |
|  |  |  | Comparative imagining of future outcomes |  | |
| Loss of contact: *immigration; separation/ divorce / death of linking relative; Estrangement*  *general loss of contact* | Environmental context and resources | *If patient doesn’t have contact with their relatives:* Suggest they ask linking person to pass information on. E.g. Their parent, aunt/uncle, or family friend may have contact with these relatives. | Instruction on how to perform the behaviour |  | |
|  |  | Discuss that some patients have been successful using the phonebook, Facebook or ancestry.com to get in touch with long-lost-relatives. Some patients have even reported BRCA to be the catalyst to contact and reunite with relatives, and it has brought their family closer. | Information about social and environmental consequences; |  | |
|  |  |  | Social support (practical); |  | |
|  |  |  | Vicarious consequences; |  | |
|  |  |  | Social comparison |  | |
|  |  | Offer to provide family letter, eviQ factsheet ‘Information for people and families with a faulty BRCA1 [or BRCA2] gene’, and Cancer Institute NSW ‘Informing family members about hereditary cancer’, so the linking relative has all the relevant resources to pass on (avoids miscommunication). | Social support (practical); |  | |
|  |  |  | Credible source; |  | |
|  |  |  | Adding objects to the environment |  | |
| Language or education barrier | Skills | *If patient feels the relative won’t comprehend the information:*  Remind the patient that it’s not their role to explain everything and stress the importance of three key messages: 1) cancer can run in the family, 2) something can be done to reduce their risk of getting cancer, and 3) provide contact details for a genetic counselling clinic. Advise that the clinic can explain the rest, as they are skilled in conveying complicated information in simple terms. Hospitals can also arrange translators. | Instruction on how to perform the behaviour; |  | |
|  |  |  | Social support (practical) |  | |
|  |  | *If patient doesn’t speak language of their relatives:*  Suggest asking a bilingual linking relative or friend to pass information on. E.g. an aunt who speaks both English and Macedonian can inform those relatives who only speak Macedonian, bridging the gap. Offer to provide family letter, eviQ factsheet ‘Information for people and families with a faulty BRCA1 [or BRCA2] gene’, and Cancer Institute NSW ‘Informing family members about hereditary cancer’ which this linking individual can use as the basis for their information. | Instruction on how to perform the behaviour; |  | |
|  |  |  | Social support (practical); |  | |
|  |  |  | Adding objects to the environment; |  | |
|  |  |  | Credible source |  | |
| Trust information was passed on by another relative | Social role^(a)^ / beliefs about consequences^(b)^ | Acknowledge that it can be difficult keeping track of the whole family, and that informing all relatives can be tiresome and emotionally laden. | Social support (emotional) | (a) | (b) |
|  |  | Encourage patient to contact informed relatives and clarify whether all relatives have been informed. E.g. Ask siblings or aunts/uncles about whether they have informed the nieces/nephews or cousins. | Instruction on how to perform the behaviour | (a) | (b) |
|  |  | Reiterate importance of ensuring all relatives have information and access to genetic counselling, and that it is often better to be told directly (or from a relative who has had genetic counselling themselves) to avoid misinformation. Explain the need to avoid situations where it is assumed that everyone has been told, yet one person doesn’t find out their BRCA risk until years later, and there is anger that they are the last one to know. Where there is concern about only seeing distant relatives at weddings or funerals (and this being an inappropriate time to raise BRCA information), explain that life events (such as death of a family member) have been shown to positively contribute to family communication about BRCA risk (Lapointe et al., 2011) as it is a time that family reflect upon their history. | Anticipated regret; | (a) | (b) |
|  |  |  | Salience of consequences; | (a) | (b) |
|  |  |  | Social comparison; | (a) | (b) |
|  |  |  | Credible source | (a) | (b) |
| Misunderstanding: *did not realise that cousins would be at risk* | Knowledge | Educate and inform the patient. Acknowledge that informing all relatives can be tiresome and emotionally laden, however the more people informed, the more lives saved. This is also an opportunity to discuss which relative may be able to pass the information on – whether cousins are directly contacted, or told by their own parent. Offer to provide family letter, eviQ factsheet ‘Information for people and families with a faulty BRCA1 [or BRCA2] gene’, and Cancer Institute NSW ‘Informing family members about hereditary cancer’ to make process easier. | Information about health consequences; |  | |
|  |  |  | Social support (emotional); |  | |
|  |  |  | Social support (practical); |  | |
|  |  |  | Adding objects to the environment; |  | |
|  |  |  | Credible source |  | |
| Misunderstanding: *patient assumes the relative will already know based on family history* | Knowledge | Explain benefits of knowing specifically about BRCA mutation risk. E.g. Relatives may be having breast screening because of a strong history of breast cancer but may be completely unaware of ovarian cancer or prostate cancer risks. Alternatively, they may be having lots of additional screening at a young age, but they may be one of the relatives who didn’t inherit the BRCA mutation; testing may relieve them of this. Provide family letter and also eviQ factsheet ‘Information for people and families with a faulty BRCA1 [or BRCA2] gene’ so patient has clear information. | Comparative imagining of future outcomes; |  | |
|  |  |  | Information about health consequences; |  | |
|  |  |  | Salience of consequences; |  | |
|  |  |  | Credible source; |  | |
|  |  |  | Adding objects to the environment |  | |
| Misunderstanding: *patient assumes BRCA mutation only affects women* | Knowledge | Educate patient that a BRCA gene mutation can be inherited by men. Explain that men have increased risks of breast and prostate cancer (and potentially pancreatic cancer in BRCA2 families), and can pass the BRCA mutation onto their children. Discuss availability of pre-implementation genetic diagnosis (PGD), and that young male relatives should be informed in case they wish to consider this option. Offer eviQ factsheet ‘Information for people and families with a faulty BRCA1 [or BRCA2] gene’ which explains implications for men. Offer Royal Marden NHS Foundation booklet ‘A Beginner’s Guide to BRCA1 and BRCA2’ which has an example letter a patient wrote to her nieces and nephews (aged 18-22) which specifically mentions PGD. | Social support (practical); |  | |
|  |  |  | Information about health consequences; |  | |
|  |  |  | Credible source; |  | |
|  |  |  | Instruction on how to perform the behaviour |  | |
| Misunderstanding: *patient assumes heritability from one side without certainty; perception often develops through family myth* | Social influences | Discuss importance of parental testing to clarify which side of the family to inform. Explain that this cannot be assumed based on the family history, providing examples of cases with unexpected BRCA lineage. Explain practical benefits – by testing one parent, it saves contacting many relatives for whom information is not relevant to and may be alarmed unnecessarily. If this is not possible, then it is important to inform both branches. Prompt patient to identify key matriarch/patriarch who they may be able to help with dissemination. | Information about health consequences; |  | |
|  |  |  | Information about social and environmental consequences; |  | |
|  |  |  | Information about emotional consequences; |  | |
|  |  |  | Social support (unspecified) |  | |

* Information and advice provided by the genetic counsellor reflects the intervention time period, and may no longer be current at time of publication

** Hodgson and Gaff (2013) suggests that in these situations counsellors need to stay focused on the patients’ emotions, rather than focusing on the consequences for the relatives. This provides greater support to the patient at hand and may be less likely to result in harm to the patient.

ACRONYMS: BCT=behaviour change technique; MoA=mechanism of action; TDF= Theoretical Domains Framework

**KEY:**

|  | **Links** | BCT-MoA link identified in both literature synthesis AND expert consensus |
| --- | --- | --- |
|  | **Non-links** | BCT-MoA link absent in literature synthesis AND experts in consensus study agreed there was no link |
|  | **Inconclusive** | Evidence of BCT-MoA link in literature synthesis but evidence of ‘no link’ in expert consensus;  OR no evidence of link (p>.05) in literature synthesis but evidence of link in expert consensus;  OR some below-criterion level of evidence in either the literature (.05< p < .10) and/or expert consensus (e.g., link agreed by 70-80% of experts) |
|  | **Absence of evidence** | No evidence of BCT-MoA link in literature synthesis, no strong evidence in expert consensus (i.e. link agreed by less than 80% of experts) |

Levels of evidence (as at 30^th^ Sept 2019) were obtained online from the Theory and Techniques Tool, available at:

<https://theoryandtechniquetool.humanbehaviourchange.org/>

A detailed explanation of the rationale and development of the tool is available from:

Carey RN, Connell LE, Johnston M, Rothman AJ, de Bruin M, Kelly MP, et al. Behavior change techniques and their mechanisms of action: a synthesis of links described in published intervention literature. Annals of Behavioral Medicine. 2018.
